# Development and internal validation of prediction models for persistence of self-reported orofacial pain in the follow-up of patients with myofascial pain

**DOI:** 10.1007/s00784-020-03570-4

**Published:** 2020-09-10

**Authors:** Naichuan Su, Frank Lobbezoo, Maurits K. A. van Selms, Geert J. M. G. van der Heijden

**Affiliations:** 1 Department of Social Dentistry, Academic Centre for Dentistry Amsterdam (ACTA), University of Amsterdam and Vrije Universiteit Amsterdam, Amsterdam, The Netherlands; 2 Department of Orofacial Pain and Dysfunction, Academic Centre for Dentistry Amsterdam (ACTA), University of Amsterdam and Vrije Universiteit Amsterdam, Amsterdam, The Netherlands

**Keywords:** Prognosis, Myofascial pain syndromes, Temporomandibular joint disorders, Risk factors

## Abstract

**Objectives:**

To identify predictors in patient profiles, and to develop, internally validate, and calibrate prediction models for the persistence of self-reported orofacial pain at the 6-month and 12-month follow-up in patients with myofascial pain.

**Materials and methods:**

A cohort of 63 adult patients with moderate to severe chronic myofascial pain was included. Patient and disease characteristics at baseline were recorded as potential predictors. Patients` presence or absence of improvement of orofacial pain at follow-up was considered the outcome. Binary logistic regression analyses were used to develop the models. The performance and clinical values of the models were determined.

**Results:**

Forty-three percent and 30% of the patients had persistence of orofacial pain at 6-month and 12-month follow-up, respectively. Pain elsewhere, depression, parafunctional activities, and mandibular function impairment (MFI) were significantly associated with persistence of the pain at 6-month follow-up, whereas depression, parafunctional activities, and MFI were significantly associated with persistence of the pain at 12-month follow-up. Both of the models showed good calibration and discrimination, with shrunken area under the curve (AUC) values of 0.73 and 0.76, respectively. The clinical added predictive values for ruling in the risk of the persistence were 0.30 and 0.31, respectively, and those for ruling it out were 0.25 and 0.20, respectively.

**Conclusions:**

Potential predictors for prediction of the persistence of self-reported orofacial pain at follow-up were identified. The calibration, discrimination, and clinical values of the models were acceptable.

**Clinical relevance:**

The models may assist clinicians in decision-making regarding the improvement of orofacial pain of individual patients during follow-up in clinical settings.

## Introduction

Temporomandibular disorders (TMD) consist of a group of disorders involving the temporomandibular joint (TMJ), masticatory muscles, or both [1]. Myofascial pain is a common subtype of TMD. In the general population, the prevalence of myofascial pain ranges from 19 to 30% [2–4], whereas among TMD patients, the prevalence of myofascial pain lies between 47 and 74% [3–6]. It is assumed that the incidence of myofascial pain is about 4% per year in the general population [4]. Based on the Research Diagnostic Criteria for TMD (RDC/TMD), myofascial pain is defined as the presence of pain history in the orofacial areas and presence of pain in temporalis or masseter muscles during clinical examination [7].

Up to 80% of the patients with complaints of myofascial pain seek for consultation to get relieved from this pain [6]. This high percentage may be due to the fact that this type of orofacial pain not only affects patients directly by hurting them physically, but the condition is also associated with a variety of psychosocial and behavioral comorbid conditions, like depression and catastrophizing. This can negatively affect patients’ daily life, activities, and work, as well as patients` interpersonal relationships with friends and families [8, 9].

To date, several treatment options are available for myofascial pain, such as counseling [10], physiotherapy [10], splint [11], psychological treatment [12], and pharmacologic treatments [13]. However, myofascial pain usually shows periods of flare-up or remission [14]. That is the most important reason that some patients show no improvement of the orofacial pain in the follow-up, whereas others show obvious improvement in the follow-up after completion of treatment [15]. For example, van Grootel et al. [16] reported that 6 and 12 out of 27 patients with myofascial pain showed no significant improvement in orofacial pain due to relapse at 6-month and 12-month follow-up after the completion of physiotherapy, respectively, whereas 5 and 8 out of 29 patients showed no significant improvement in orofacial pain due to relapse in 6-month and 12-month follow-up after the completion of splint, respectively. This may indicate that some patients are more likely to have no improvement in complaints at follow-up compared with others. Therefore, more knowledge is needed to identify what type of patients with myofascial pain is more likely to have persistence of orofacial pain in the follow-up. This may provide clinicians with important information for their decision-making for the management of individual patients in health care at follow-up.

The aims of the present study were to (1) identify potential predictors in patient profiles that allow prediction of the persistence of self-reported orofacial pain in the follow-up in patients with myofascial pain; and (2) develop, internally validate, and calibrate models for prediction of the persistence of the pain during follow-up.

## Materials and methods

The TRIPOD (Transparent Reporting of a multivariable prediction model for Individual Prognosis Or Diagnosis) statement [17] was followed for the development of the models.

### Study design

The study was designed as a cohort study with patients’ self-reported chronic orofacial pain scores as the observational outcomes during a 12-month follow-up. Each patient received a total of six questionnaires at six predetermined time points. The questionnaires were given at patients’ first visit to the clinic and directly after completion of treatment. Then other questionnaires were sent to the patients at 3, 6, 9, and 12 months after the completion of treatment by mail. Patients were reminded by receiving a telephone call or a mail if the questionnaires were not returned within 2 to 3 weeks.

The cohort at baseline involved 63 patients with complaints of pain in the orofacial region lasting for at least 1 month, who were referred by their dentist or medical practitioner to the clinic for Orofacial Pain and Dysfunction of Academic Centre for Dentistry Amsterdam (ACTA) between October 2003 and November 2006.

### Participant enrolment

The inclusion criteria for the patients at their first visit to the clinic were as follows: (1) Patients were diagnosed with myofascial pain according to the RDC/TMD [7]; (2) Patients were over 18 years of age; (3) Patients had no systemic disease and no other orofacial disorders like trigeminal neuralgia; (4) Patients had no severe psychiatric disorders; (5) Patients had no overuse of painkillers; (6) Patients had a good understanding of the Dutch language; and (7) Patients reported a moderate to severe orofacial pain score at baseline with the score of Characteristic Pain Intensity (CPI) scale ≥ 35 (The range of CPI score is 0–100) [18].

The patients were treated by clinicians experienced in the management of TMD patients. The provided conservative (i.e., non-invasive) treatments included counseling, occlusal splint, physiotherapy, cognitive behavior modification therapy, or combinations of these treatment modalities.

The decision to end the treatment was based on the assessment of whether further treatment would be of substantial benefit to the patient. The initial treatments indicated for patients were proposed by the clinician who performed the intake examination. For uncomplicated patients, the type of treatment indicated for patients was discussed between the clinician who performed the intake examination and a senior consultant for a final decision. More complex patients were discussed in the multidisciplinary team (including senior consultants, dentists, physiotherapists, and a psychologist), and the staff represented in the multidisciplinary team made the final decisions together. The final decision-making of the senior consultants and the multidisciplinary team was based on their expertise, experience, and knowledge as well as on patients` specific signs and symptoms in both physical and psychological aspects. Whether patients were regarded as uncomplicated or complicated was judged by clinicians based on their clinical expertise, experience, and knowledge as well as on patients` specific signs and symptoms.

### Potential predictors

Patients’ demographic characteristics, including gender and age, were recorded at baseline as predictors. Besides, the following predictors were also recorded at baseline as the potential predictors of the models (Table 1):

1. Pain elsewhere in the body was recorded with the question “Do you feel pain elsewhere in your body?” The answer was classified as “no” or “yes.”
2. Previous treatment for complaints of TMD pain was recorded. The answer was classified as “no” or “yes.”
3. Somatization over the past week was assessed with the somatization scale of the Dutch version of the Symptom Check List (SCL-90) [19, 20]. The somatization scale assesses the extent that patients are bothered by distress related to bodily symptoms, including faintness and stomach upset. The somatization scale of SCL-90 included 12 items. Each item is answered on a 5-point Likert scale ranging from not at all (1) to very much (5). In the present study, a shortened somatization scale, excluding the four pain-related questions (headache, pain in the chest or heart region, pain in the lower back, and painful muscles), was used to avoid confounding with questions assessing pain throughout the body. Therefore, only 8 items of the somatization scale were included in the present study, and the sum score of the shortened somatization scale ranges from 8 to 40; higher scores indicate more severe somatic symptoms.
4. Depression over the past week was assessed with the depression scale of the Dutch version of the SCL-90 [19, 20]. The depression scale assesses the extent that patients are bothered by negative mood and vegetative symptoms of poor functioning. The depression scale of SCL-90 includes 16 items, and each item is answered on a 5-point Likert scale ranging from not at all (1) to very much (5). The sum score of the depression scale ranges from 16 to 80, with higher scores indicating more severe depressive symptoms.
5. Pain chronicity (duration of TMD pain complaints) of each patient was recorded in months.
6. Parafunctional activities of patients in the past 3 months were assessed with a three-scale oral parafunction questionnaire [15, 21]. The first scale of this questionnaire involved four items related to clenching and grinding at night or during the day. The second scale involved three items related to biting and chewing activities. The third scale involved five items related to tongue, lip, and cheek activities. Each item is answered on a 5-point Likert scale ranging from never (0) to always (4). The sum score is ranging from 0 to 48. The total parafunctional activity score was calculated by dividing the sum score by 48. Therefore, the total parafunctional activity score was ranging from 0 to 1, with higher scores indicating more severe parafunctional activities.
7. Patients’ chronic orofacial pain intensity was assessed with the CPI scale [22]. The CPI score ranges from 0 to 100 and is assessed by calculating the mean of current facial pain intensity (0 to 10), the average facial pain intensity (0 to 10), and the worst facial pain intensity (0 to 10) in the last 3 months, and by multiplying this score by 10. Higher CPI scores indicate higher orofacial pain intensity.
8. Patients’ mandibular function impairment was assessed with the Mandibular Function Impairment Questionnaire (MFIQ) [23]. The MFIQ assesses how much difficulty patients experience when performing a particular mandibular task. The MFIQ consists of a total of 17 items, and each item is answered on a 5-point Likert scale ranging from 0 (no difficulty) to 4 (very difficult or impossible without help). The MFI score ranges between 0 and 1 and is obtained by dividing the sum scores of the items by 68. Higher MFI scores indicate more severe mandibular function impairment.

**Table 1:** Distribution of the potential predictors for prediction of persistence of orofacial pain at 6-month and 12-month follow-up (*N* = 63)

Variables	No. of patients (*N* = 63)	CPI^a^ at 6-month follow-up (model 1)		CPI^a^ at 12-month follow-up (model 2)	
		No. of patients with improvement (*N* = 36)	No. of patients with persistence (*N* = 27)	No. of patients with improvement (*N* = 44)	No. of patients with persistence (*N* = 19)
Age	40.5 ± 13.9	40.1 ± 13.9	41.0 ± 14.05	40.1 ± 14.4	41.2 ± 12.9
Gender					
Female	55	31	24	40	15
Male	8	5	3	5	4
Pain elsewhere					
No	33	23	10	24	9
Yes	30	13	17	20	10
Previous treatment					
No	26	14	12	16	10
Yes	37	22	15	28	9
Somatization	12.5 ± 5.8	11.4 ± 5.1	14.0 ± 6.5	12.4 ± 5.7	12.9 ± 6.3
Depression	22.2 ± 6.9	20.3 ± 5.7	24.6 ± 7.7	21.0 ± 6.3	25.0 ± 7.5
Pain chronicity (month)	44.8 ± 52.8	43.6 ± 58.1	46.5 ± 46.0	41.2 ± 52.5	53.4 ± 53.3
Parafunctional activities	0.24 ± 0.15	0.21 ± 0.13	0.28 ± 0.16	0.22 ± 0.13	0.30 ± 0.17
CPI^a^	60.6 ± 15.5	61.6 ± 16.7	59.4 ± 13.9	60.9 ± 15.9	59.8 ± 14.5
MFI^b^	0.42 ± 0.21	0.43 ± 0.20	0.39 ± 0.23	0.44 ± 0.21	0.34 ± 0.21

^a^Characteristic Pain Intensity; ^b^Mandibular Function Impairment

### Outcomes

Changes in orofacial pain intensity at 6-month or 12-month follow-up compared with the baseline were regarded as the outcome for the two models. The outcome was binary. In the present study, if a patient’s CPI score did not decrease by 37.9% or more at follow-up compared with the CPI score at baseline, the orofacial pain of the patients in the follow-up was considered to be “persistent.” Otherwise, orofacial pain was considered to be “improved.” This cutoff was based on the study conducted by Emshoff et al. [24]. This cutoff was selected because the clinically important change (CIC) on a 100-mm visual analog scale for pain intensity at this cutoff was best associated with the pre-defined concept of CIC based on the commonly used and validated measure of the patient`s global impression of change (PGIC) [24]. Because patients with high baseline pain required greater absolute reductions in pain to reach a clinically important improvement, relative change scale performed better in classifying improved patients than absolute changes scale.

### Statistical analysis

#### Missing data

Multiple imputation technique was used to handle the missing values via SPSS software 25 (IBM, New York, USA). We created *m* = 30 imputed datasets with 10 iterations and used predictive mean matching (PMM) for imputing the missing values. All the potential predictors, the CPI score directly after completion of treatment, and the CPI scores at 3, 6, 9, and 12 months after the completion of treatment were included in the imputation model.

#### Modeling

Multivariable binary logistic regression analysis with backward-selection (predictors with *P* > 0.25 were removed from the models and predictors with *P* ≤ 0.25 remained in the model) was used to assess the association of potential predictors with the outcomes and to develop the prediction models. A less stringent threshold of *P* value of 0.25 was used in selection and exclusion of potential predictors because this could avoid that some important predictors were excluded inappropriately from the models due to large *P* values caused by small sample size [25].

#### Shrinkage factor

Regression models developed and tested in the same (derivation) dataset not always provide fully accurate predictions. That is, higher predictions will be found too high while low predictions will be found too low [26]. Therefore, shrinkage techniques were proposed as a remedy against too extreme predictions. The simplest method in shrinkage techniques is to shrink the intercept and regression coefficients of the models by a common factor, which is called shrinkage factor [26]. A shrinkage factor can be produced from bootstrapping techniques and ranges from 0 to 1. Shrinkage factor can prevent for the overfitting of the current model that has been developed from a derivation dataset and for over-optimism of a model applied in similar future patients [27, 28]. The shrinkage factor in this study was derived using the bootstrapping procedure with 10 bootstrapping samples.

#### Calibration

Calibration is defined as the agreement between predicted outcomes and observed outcomes [29]. It reflects the extent to which a model correctly estimates the absolute risk [30]. The calibration of the models was assessed by plotting the predicted individual outcomes against the observed actual outcomes. For this, study members were grouped into deciles based on their predicted probability for the persistence of orofacial pain at follow-up separately. The prevalence of the endpoint within each decile represents the observed probability. In the calibration plot, the observed and predicted probabilities were compared across the range of predicted risks. The calibration of the multivariate models was also assessed using the Hosmer-Lemeshow goodness-of-fit statistic test (HL test). A *P* value of > 0.10 in the HL test indicates that the model fits the observed data [31].

#### Discrimination

Discrimination is defined as the ability to differentiate between those with and those without the outcome event [29]. The outcome event in the present study was the persistence of orofacial pain. The area under the receiver-operating characteristic curve (AUC) was used to assess the discrimination of the models [32]. An AUC of 0.70 to 0.80 indicates that the discrimination of the prediction model is acceptable, whereas an AUC of ≥ 0.80 indicates that the discrimination of the prediction model is excellent to outstanding [33].

The optimal cutoff for the predicted probability of the models was defined as the predicted probability with the maximum sum of sensitivity and specificity in the receiver-operating characteristic curve (ROC).

#### Clinical values

Clinical values of the models at the optimal cutoffs for predicted probability were assessed with the prevalence, positive predictive values (PPV), and negative predictive values (NPV) of patients with persistence of orofacial pain at follow-up. PPV was defined as the number of patients with persistence of orofacial pain as observed in reality in patients with persistence of orofacial pain as predicted by the models. NPV was defined as the number of patients with improvement as observed in reality in patients with improvement as predicted by the models. The (added) predictive value of the models at the certain cutoff for predicted probability for ruling in an increased risk in the persistence of orofacial pain at the follow-up was defined as PPV minus prevalence, while that for ruling out an increased risk in the persistence of orofacial pain was defined as NPV minus complement of prevalence.

#### Scoring system

We developed a clinical rule for prediction of the persistence of orofacial pain in the present study as to provide an estimate for individual patients of their absolute risk of the persistence of orofacial pain at the follow-up. For the final multivariate logistic regression models, the probability (*P*) of persistence of orofacial pain is predicted with the following formula:

$$ P=1-1/\left[1+\exp \left(\mathrm{constant}+\upbeta 1\mathrm{X}1+\dots +\upbeta \mathrm{iXi}\right)\right] $$

where *β* is the shrunken regression coefficient of a predictor in the models.

To facilitate the calculation of the predicted outcomes in individual patients separately, the multivariable logistic regression models were converted to a score chart. The scores of each included predictor in the score chart were produced by the shrunken regression coefficients. Then, the models were transformed into line charts. The *X*-axis of the line charts represents the total scores of the individual patients, whereas the *Y*-axis represents the predicted probability for the persistence of orofacial pain of individual patients.

Multivariable binary logistic regression analysis with backward-selection, shrinkage factor, discrimination, calibration, clinical values, and scoring system of the models were all produced based on the 30 imputed datasets via R software 3.4.3 (R Development Core Team, Vienna, Austria) using the package “psfmi.” The discrimination, calibration, and scoring system of the models were assessed based on the shrunken regression coefficients.

### Sample size estimation

For binary logistic regression analyses, the prediction model is considered reliable if the number of events per variable (EPV) is ≥ 10 [34, 35]. The total number of variables is calculated as the number of the continuous predictors plus the number of categories (without the reference category) for categorical predictors in the multivariate binary logistic regression analyses.

## Results

At baseline, a total of 63 patients were included in the analyses. Of these, 55 (87%) were female. The mean age was 40.4 ± 14.0 for women and 40.6 ± 14.2 for men. There were 51 and 45 patients included in the study at 6-month and 12-month follow-up, respectively. Forty-five percent (23/51) and 28% (13/45) of the patients were documented as persistence of orofacial pain at 6-month and 12-month follow-up, respectively.

**Fig. 1:**
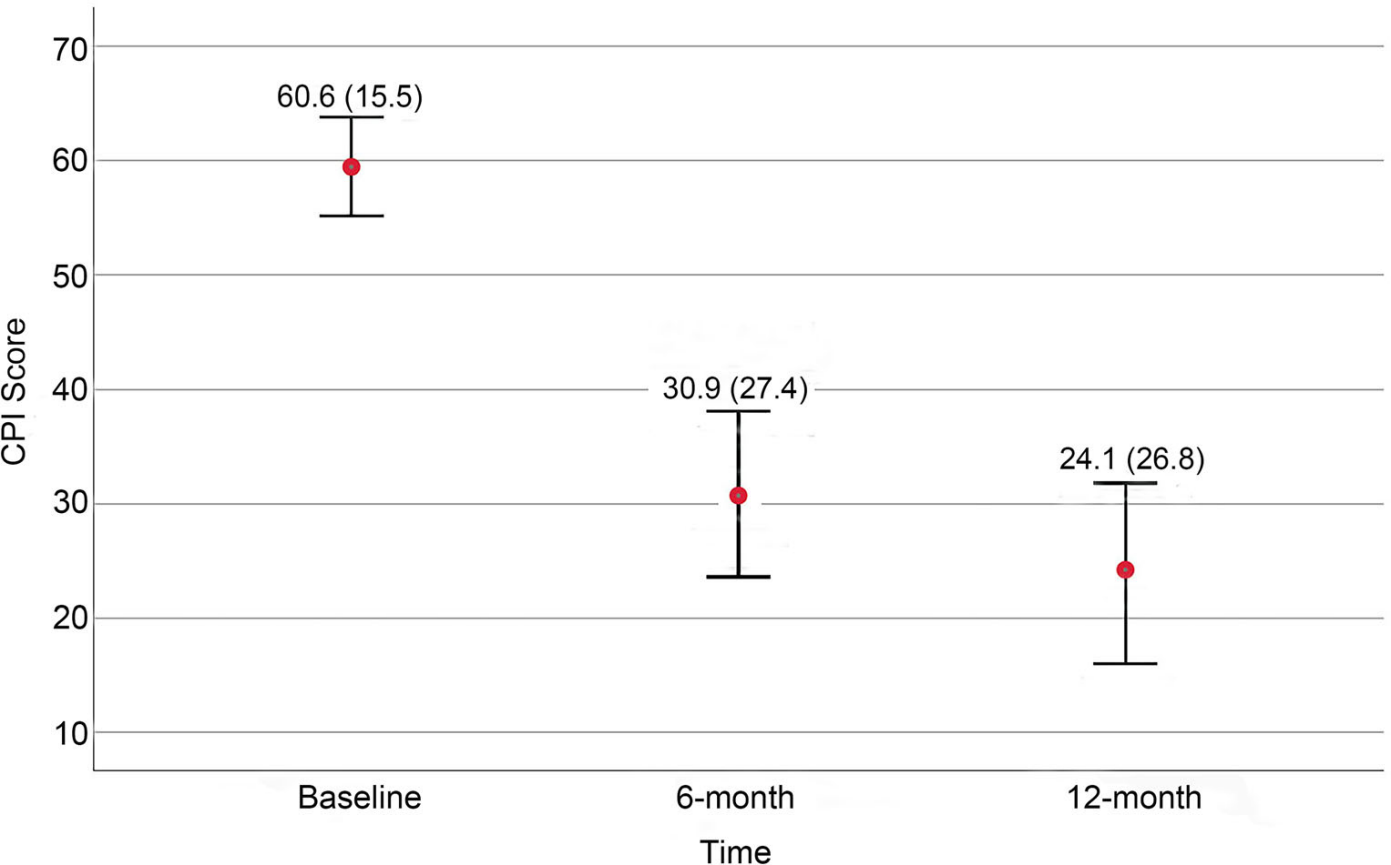
The mean scores (standard deviations) of the Characteristic Pain Intensity (CPI) scores at baseline, 6-month, and 12-month follow-up

As for the missing values, MFI at baseline, the outcome variable at 6-month follow-up, and the outcome variable at 12-follow-up were missing in 4, 12, and 18 patients, respectively, while the remaining variables had no missing values. After multiple imputation, 43% (27/63) and 30% (19/63) of the patients had the persistence of orofacial pain at 6-month and 12-month follow-up, respectively. Table 1 presents the distribution of baseline predictors based on the multiple imputation. Figure 1 shows the mean scores of the CPI at baseline, at 6-month, and at 12-month follow-up based on the multiple imputation.

Table 2 shows the predictors included in the final logistic multivariate models based on backward-selection (*P* > 0.25 with removal) for prediction of the persistence of orofacial pain at 6-month and 12-month follow-up. In the multivariate analysis, patients with the presence of pain elsewhere, more depression, more parafunctional activities, and less mandibular function impairment at baseline were more likely to have persistence of orofacial pain at 6-month follow-up, whereas patients with more depression, more parafunctional activities and less mandibular function impairment at baseline were more likely to have persistence of orofacial pain at 12-month follow-up.

**Table 2:** Multivariate binary logistic regression analyses (using backward selection procedures, *P* ≤ 0.25) of the potential predictors for prediction of the persistence of orofacial pain at 6-month and 12-month follow-up (*N* = 63)

	CPI^b^ at 6-month follow-up (model 1) (*N* = 63)			CPI^b^ at 12-month follow-up (model 2) (*N* = 63)		
	*Β*^d^ (SE^e^)	Shrunken *B*^d^	OR^f^ (95% CI)	*Β*^ad^ (SE^e^)	Shrunken *B*^d^	OR^f^ (95% CI)
Pain elsewhere						
No	Reference					
Yes	1.287 (0.638)	1.046	3.62 (1.04 12.65)			
Depression	0.098 (0.051)	0.080	1.10 (1.00 1.22)	0.089 (0.063)	0.079	1.09 (0.97 1.24)
Parafunctional activities	2.893 (2.102)	2.351	18.04 (0.29 1112.33)	3.983 (2.514)	3.548	53.70 (0.38 7515.66)
MFI^c^ at baseline	− 1.932 (1.602)	− 1.571	0.14 (0.01 3.37)	− 3.515 (1.997)	− 3.131	0.03 (0.00 1.53)^a^
Constant	− 3.037 (1.343)	− 2.512		− 2.557 (1.500)	− 2.359	

^a^The exact OR and 95% CI is 0.0297 (0.0006 1.5255); ^b^Characteristic Pain Intensity; ^c^Mandibular Function Impairment; ^d^Regression coefficient; ^e^Standard error; ^f^Odds ratio

**Fig. 2:**
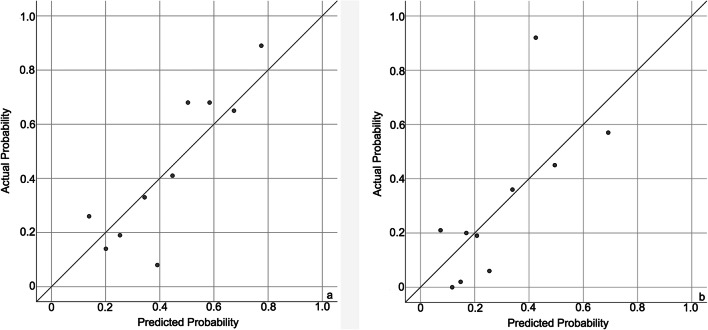
Calibration plots of the multivariate logistic regression models for the persistence of orofacial pain at 6-month (**a**) and 12-month (**b**) follow-up. **a** The calibration plot of the improvement of orofacial pain at 6-month follow-up (The number of patients with actual persistence is 27 while that with predicted improvement is 29). **b** The calibration plot of the improvement of orofacial pain at 12-month follow-up (The number of patients with actual improvement is 19 while that with predicted improvement is 23). The diagonal represents the predicted probability of the model is the same as the actual probability of the model so that the prediction is neither underestimated nor overestimated. The dots represent the deciles of the study members based on their predicted probability

The shrinkage factors of the models for persistence of orofacial pain at 6-month and 12-month follow-up were 0.81 and 0.89, respectively. The AUCs of the models at 6-month and 12-month follow-up were 0.77 (95% confidence interval (CI): 0.50 0.92) and 0.78 (95% CI: 0.47 0.94), respectively. The shrunken AUCs of the models at 6-month and 12-month follow-up were 0.73 and 0.76, respectively, which indicated that the discrimination of the two models was acceptable. The calibration plots showed that there was a good fit between the predicted probability and actual probability of the persistence of orofacial pain in both models, considering that most plotted dots in both models were lying close to the diagonal line (Fig. 2). With resulting values for the HL tests of 0.74 and 0.80, the two models were shown to have good fit.

The cutoffs for the predicted probability of the persistence of orofacial pain at 6-month and 12-month follow-up were 0.46 and 0.31, respectively. Table 3 presents the prevalence, sensitivity, specificity, PPV, NPV, the added values for ruling in the patients with persistence of orofacial pain in addition to the prevalence, and the added values for ruling out the patients with the persistence in addition to the complement of the prevalence of the two models at the cutoffs for the predicted probability.

**Table 3:** Clinical values of the models for prediction of persistence of orofacial pain at 6-month and 12-month follow-up (*N* = 63)

Models	Prevalence	Sensitivity	Specificity	PPV^b^	NPV^c^	Added value for ruling in the outcome	Added value for ruling out the outcome
CPI^a^ at 6-month follow-up (model 1)	0.43	0.76	0.78	0.72	0.81	0.30	0.25
	(0.31 0.55)	(0.59 0.90)	(0.62 0.89)	(0.54 0.86)	(0.67 0.93)	(0.10 0.50)	(0.08 0.43)
CPI^a^ at 12-month follow-up (model 2)	0.30	0.77	0.81	0.63	0.89	0.31	0.20
	(0.20 0.42)	(0.51 0.90)	(0.68 0.91)	(0.40 0.79)	(0.78 0.97)	(0.11 0.50)	(0.06 0.35)

^a^Characteristic Pain Intensity; ^b^Positive predictive value; ^c^Negative predictive value

**Fig. 3:**
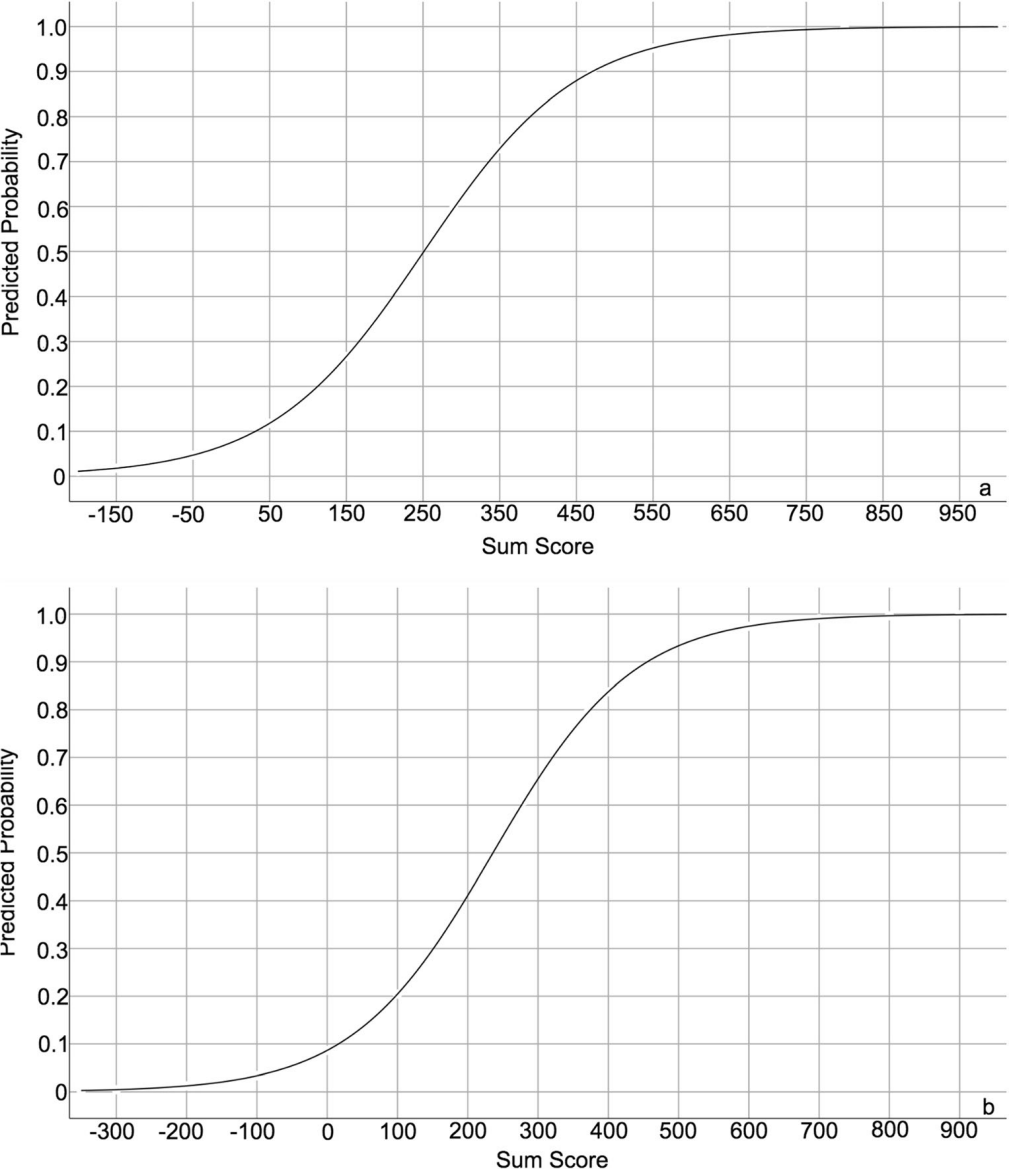
The line charts of the multivariate logistic regression models for the persistence of orofacial pain at 6-month (**a**) and 12-month (**b**) follow-up. From the line charts, the exact predicted probability (%) of the persistence of orofacial pain at follow-up for an individual (axis *Y*) can be determined based on the sum scores (axis *X*) and the curves

To enhance the clinical usefulness of the models, we transformed the final regression models into a score chart (Table 4) and two line charts (Fig. 3) based on the shrunken regression coefficients. Based on the score chart, a clinician can easily calculate the sum scores of individuals for prediction of the persistence of orofacial pain at both 6-month and 12-month follow-up. Then, a clinician can determine the corresponding predicted probability of the persistence based on their sum scores by using Fig. 3. The cutoffs of the sum scores of the two models were 237 and 154, respectively.

**Table 4:** Score chart of the models for prediction of the persistence of orofacial pain at 6-month and 12-month follow-up

	CPI^b^ at 6-month follow-up (model 1)		CPI^b^ at 12-month follow-up (model 2)
Predictors	Score	Predictors	Score
Pain elsewhere		Depression	8*( )
No	0	Parafunctioal activities	355*( )
Yes	105		
Depression	8*( )	MFI^a^ at baseline	− 313*( )
Parafunctional activities	235*( )		
MFI^a^ at baseline	− 157*( )		
Sum score at 6-month follow-up	( )	Sum score at 12-month follow-up	( )

^a^Mandibular Function Impairment; ^b^Characteristic Pain Intensity

Instructions: The score of each predictor can be calculated as the given number provided in the table for each predictor multiplied by the score that the patient gets for this predictor. The sum score is the sum of the scores of each predictor in the table

For example, if a patient has a pain elsewhere in the body, a score of 20 for depression, a score of 0.5 for parafunctional activities, and a score of 0.4 for MFI at baseline, the sum score of the model at 6-month follow-up of this patient based on Table 4 can be calculated as 105 + 8*20 + 235*0.5 − 157*0.4 = 319.7, whereas the sum score of the model at 12-month follow-up is 8*20 + 355*0.5 − 313*0.4 = 212.3. The sum score of this patient at 6-month follow-up is larger than the cutoff of the sum score (237), so this patient is likely to have persistence of orofacial pain at 6-month follow-up. The sum score of this patient at 12-month follow-up is also larger than the cutoff of the sum score (154), so this patient is also likely to have persistence of orofacial pain at 12-month follow-up. Based on Fig. 3, the predicted probability of having the persistence of this patient at 6-month and 12-month follow-up is around 66% and 44%, respectively.

## Discussion

The present study has shown that patients with the presence of pain elsewhere, more depression, more parafunctional activities, and less severe mandibular function impairment at baseline were more likely to have persistence of orofacial pain at 6-month follow-up. Patients with more depression, more parafunctional activities, and less severe mandibular function impairment at baseline were more likely to have persistence of orofacial pain at 12-month follow-up.

Depression was an important predictor for the persistence of orofacial pain at both 6-month and 12-month follow-up. When patients’ score of depression based on the Dutch version of SCL-90 was increased by 1 unit, the odds of having persistence of orofacial pain at 6-month follow-up will be increased by 10%, whereas the odds of having persistence of orofacial pain at 12-month follow-up will be increased by 9%. Depression can disrupt the pathways between central and peripheral nerves, thus affecting the descending inhibitory pathways. The modulation of pain through these descending pathways is disrupted, and pain sensations are allowed to ascend to the brain [36]. It is reported that patients with chronic pain and comorbid mood disorders are often more difficult to treat, because of a difference in perception of pain, negative coping skills, the risk of drug abuse, and the negative influence of comorbidity on the outcomes of diagnosis [37, 38]. That may explain why among patients with more severe depression at baseline, it was more difficult to get relieved from their orofacial pain in the follow-up after treatment. Besides, patients with less severe mandibular function impairment at baseline were more likely to have persistence of orofacial pain at both 6-month and 12-month follow-up. One of the reasons might be that the orofacial pain in those patients with less severe mandibular function impairment was more psychosocially related rather than physically or functionally related. Psychosocially related pain is often more difficult and challenging to treat than other types of pain [39]. Patients with more severe parafunctional activities at baseline were more likely to have persistence of orofacial pain at both 6-month and 12-month follow-up. That may be because persons with more severe orofacial pain have a tendency to overestimate their parafunctional activities [15]. Besides, parafunctional activities, such as awake and sleep bruxism, may result in orofacial pain, to some extent [40]. Therefore, even though the orofacial pain of patients with parafunctional activities was relieved after treatment, their orofacial pain was likely to relapse in the follow-up if they still keep the parafunctional activities.

However, it should be noted that the predictors included in the prediction models are significantly associated with the outcomes, but it is irrespective of causality. That is, perhaps not all of the predictors in the models have a causal relationship with outcomes and a variable may be a strong predictor even if it has no causal relationship with outcomes.

So far, several studies have assessed the different factors from different domains as predictors for the pain-related outcomes in TMDs. For example, Osiewicz et al. [41] assessed the association of biological, psychological, and social factors with the presence of painful TMDs, and found that depression was the most important predictor for the presence of painful TMD. Banafa et al. [42] assessed the association of socio-demographic background and denture status at baseline with TMJ pain and masticatory muscles pain on palpation after 11-year follow-up in Finnish adult population, and found that all the predictors, including gender and age, were not significantly associated with the presence of TMJ pain at follow-up. Kapos et al. [43] assessed the association of health-related quality of life and jaw functional limitation at baseline with long-term pain intensity of TMDs at 8-year follow-up, and found that higher CPI scores and higher physical health-related quality of life at baseline were significantly associated with the lower TMD pain intensity at 8-year follow-up. Most of the findings of these previous studies were consistent with our findings in the present study. However, all the studies mentioned above were only aimed at finding out the significant variables rather than developing prediction models for the pain-related outcomes in TMDs. Up to date, only a small number of prediction models have been developed on TMDs for the prediction of different outcomes, for example, the persistence of TMDs, oral health-related quality of life, and types of treatment indicated for patients with TMDs [44–46]. However, no study has focused on the development of prediction models for pain-related outcomes in TMDs so far.

Based on common and easily obtainable clinical variables mentioned above, we performed multivariate logistic models to predict the persistence of orofacial pain in patients with myofascial pain at follow-up. With the prediction models in the present study, clinicians can easily predict whether or not the patients have the persistence of orofacial pain at 6-month and 12-month follow-up. If patients are predicted to be very likely to have persistence of orofacial pain at the follow-up, clinicians can pay more attention to these patients, consider to do re-evaluations regularly, and give additional treatment at appropriate time points. Besides, the reported models may help to inform patients on their prognosis in the follow-up, and shape their expectations for the prognosis of the disease.

To facilitate the use of the prediction models in clinical practice, it is important to determine the optimal cutoff for predicted probability for probability stratification. It is the point at which the sum of sensitivity and specificity is at its maximum, and where misclassification is lowest. The present models regarded 0.46 and 0.31 as the cutoffs for predicted probability of persistence of orofacial pain at the 6-month and 12-month follow-up, respectively. Hence, when the sum scores of individual patients were higher than 237 and 154 in the two models, respectively, patients were most accurately predicted to have persistence in orofacial pain at the follow-up.

Notwithstanding the above, it should be noted that prediction models may cause false negative and false positive predictions. With false negative predictions, a patient who may actually need more frequent follow-ups after treatment, and/or more extra treatment in the follow-up, is unlikely to receive this, which may exacerbate the disease and cause less desired health outcomes. In contrast, with false positive predictions, a patient who actually may not need more treatments or regular follow-up after treatment is likely to receive this anyway. Therefore, this may give rise to an increase in the financial and psychological burden, and a waste of time and resources. Based on the present models, the risk of a false positive prediction of persistence at 6-month and 12-month follow-up was both 13%, while the risk of a false negative prediction of the two models was 10% and 7%, respectively. This indicated that the risk for both false positives and false negatives can be considered small and acceptable.

The added predictive values of the two models for ruling in the persistence of orofacial pain at 6-month and 12-month follow-up were 0.30 and 0.31, respectively, while those for ruling it out were 0.25 and 0.20, respectively. This indicates that if a patient is predicted to have persistence of orofacial pain at 6-month and 12-month follow-up based on the models, the posterior probability of having persistence of orofacial pain of this patient can be increased by 0.30 and 0.31 compared with the prevalence (prior probability) of persistence of orofacial pain in the patients group. If a patient is predicted to have an improvement in the follow-up based on the models, the posterior probability of the improvement in the follow-up of this patient can be increased by 0.25 and 0.20, respectively, when compared with the prevalence of improvement of orofacial pain in the patients group. This indicated that the clinical added values of the two models were sufficient for both ruling in and out the persistence of orofacial pain at follow-up.

Only patients with a moderate to severe orofacial pain were included in the development of the prediction models. This was because in case patients with myofascial pain only have very mild or even no obvious self-reported chronic orofacial pain at baseline, there is almost no ability to have a significant improvement in orofacial pain in the follow-up. Those patients are very likely to be classified into the event group incorrectly even though they are not bothered by pain, thus making the models biased. Therefore, the two models in the study can only be applicable for patients with myofascial pain with moderate to severe self-reported chronic orofacial pain at baseline.

A smaller number of events relative to the high number of predictors are a common limitation for multivariable prediction models. The present study did not meet the criterion of EPV ≥ 10 [34, 35] because of the small sample size, which is a major limitation of the study. This may yield biased estimates of regression coefficients and lead to the overfitting of the models especially in the absence of external validation. Therefore, clinicians should be cautious about the predicted results from the models when they make decisions in clinical practice. Due to the small sample size, a less stringent threshold of *P* value of 0.25 was used in modeling for selection and exclusion of potential predictors. However, in order to lower the risk that some unimportant predictors were included inappropriately in the model when the cutoff of *P* value is increased, all the potential predictors were pre-screened by clinicians based on their clinical experience and knowledge before data analysis. Therefore, in this way, the negative consequence caused by small sample size could be reduced to a large extent.

Another limitation of the study is that the models were not developed based on the most recent data. Instead, the data were obtained between 2003 and 2006. Changes in practice over time can limit the application of the prediction models [39]. For example, modifications in diagnostic criteria from RDC/TMD to Diagnostic Criteria for TMD (DC/TMD) [47] and improvements in treatments may change the prognosis of the patients. Therefore, this may lead to the situation that the developed prediction models in the present study, based on old data, cannot have a satisfactory predictive ability for current patients.

In the future, researchers are suggested to use a larger sample size to externally validate and update the models based on the most recent samples of patients with myofascial pain before the models can be applied in clinical practice.

## Conclusions

Pain elsewhere, depression, parafunctional activities, and mandibular function impairment at baseline were important predictors for the persistence of orofacial pain at 6-month follow-up, whereas depression, parafunctional activities, and mandibular function impairment at baseline were important predictors for the persistence of orofacial pain at 12-month follow-up. The multivariate logistic regression models for prediction of persistence of orofacial pain at follow-up were developed and internally validated. The added predictive values may be sufficient for both ruling in and ruling out the persistence of orofacial at follow-up. The models may assist clinicians in decision-making regarding the improvement of orofacial pain of individual patients during follow-up in clinical settings. External validation of the model is needed before considering the implementation of the model in clinical practice.
